# Effects of Different Levels of Variability and Pressure Support Ventilation on Lung Function in Patients With Mild–Moderate Acute Respiratory Distress Syndrome

**DOI:** 10.3389/fphys.2021.725738

**Published:** 2021-10-22

**Authors:** Lorenzo Ball, Yuda Sutherasan, Martina Fiorito, Antonella Dall'Orto, Lorenzo Maiello, Maria Vargas, Chiara Robba, Iole Brunetti, Davide D'Antini, Pasquale Raimondo, Robert Huhle, Marcus J. Schultz, Patricia R. M. Rocco, Marcelo Gama de Abreu, Paolo Pelosi

**Affiliations:** ^1^Department of Surgical Sciences and Integrated Diagnostics, University of Genoa, Genoa, Italy; ^2^Anesthesia and Intensive Care, Ospedale Policlinico San Martino Istituto di Ricerca e Cura a Carattere Scientifico (IRCCS) for Oncology and Neurosciences, Genova, Italy; ^3^Division of Pulmonary and Pulmonary Critical Care Medicine, Department of Medicine, Ramathibodi Hospital, Mahidol University, Bangkok, Thailand; ^4^Department of Neurosciences, Reproductive and Odonthostomatological Sciences, University of Naples Federico II, Naples, Italy; ^5^Department of Anaesthesia and Intensive Care, University of Foggia, Foggia, Italy; ^6^Mahidol Oxford Tropical Medicine Research Unit (MORU), Mahidol University, Bangkok, Thailand; ^7^Department of Intensive Care, Laboratory of Experimental Intensive Care and Anesthesiology (LEICA), Amsterdam University Medical Centers, Location Academic Medical Center (AMC), Amsterdam, Netherlands; ^8^Nuffield Department of Medicine, Oxford University, Oxford, United Kingdom; ^9^Laboratory of Pulmonary Investigation, Carlos Chagas Filho Institute of Biophysics, Federal University of Rio de Janeiro, Rio de Janeiro, Brazil; ^10^Pulmonary Engineering Group, Department of Anaesthesiology and Intensive Care Medicine, University Hospital Carl Gustav Carus, Technische Universität Dresden, Dresden, Germany

**Keywords:** variable pressure support ventilation, acute respiratory distress (ARDS), asynchronies, respiratory mechanic, assisted ventilation

## Abstract

**Background:** Variable pressure support ventilation (vPSV) is an assisted ventilation mode that varies the level of pressure support on a breath-by-breath basis to restore the physiological variability of breathing activity. We aimed to compare the effects of vPSV at different levels of variability and pressure support (Δ*P*_S_) in patients with acute respiratory distress syndrome (ARDS).

**Methods:** This study was a crossover randomized clinical trial. We included patients with mild to moderate ARDS already ventilated in conventional pressure support ventilation (PSV). The study consisted of two blocks of interventions, and variability during vPSV was set as the coefficient of variation of the Δ*P*_S_ level. In the first block, the effects of three levels of variability were tested at constant Δ*P*_S_: 0% (PSV_0%_, conventional PSV), 15% (vPSV_15%_), and 30% (vPSV_30%_). In the second block, two levels of variability (0% and variability set to achieve ±5 cmH_2_O variability) were tested at two ΔP_S_ levels (baseline Δ*P*_S_ and Δ*P*_S_ reduced by 5 cmH_2_O from baseline). The following four ventilation strategies were tested in the second block: PSV with baseline Δ*P*_S_ and 0% variability (PSV_BL_) or ±5 cmH_2_O variability (vPSV_BL_), PSV with ΔP_S_ reduced by 5 cmH_2_O and 0% variability (PSV_−5_) or ±5 cmH_2_O variability (vPSV_−5_). Outcomes included gas exchange, respiratory mechanics, and patient-ventilator asynchronies.

**Results:** The study enrolled 20 patients. In the first block of interventions, oxygenation and respiratory mechanics parameters did not differ between vPSV_15%_ and vPSV_30%_ compared with PSV_0%_. The variability of tidal volume (*V*_T_) was higher with vPSV_15%_ and vPSV_30%_ compared with PSV_0%_. The incidence of asynchronies and the variability of transpulmonary pressure (*P*_L_) were higher with vPSV_30%_ compared with PSV_0%_. In the second block of interventions, different levels of pressure support with and without variability did not change oxygenation. The variability of *V*_T_ and *P*_L_ was higher with vPSV_−5_ compared with PSV_−5_, but not with vPSV_BL_ compared with PSV_BL_.

**Conclusion:** In patients with mild-moderate ARDS, the addition of variability did not improve oxygenation at different pressure support levels. Moreover, high variability levels were associated with worse patient-ventilator synchrony.

**Clinical Trial Registration:** www.clinicaltrials.gov, identifier: NCT01683669.

## Introduction

Pressure support ventilation (PSV) is an assisted ventilation mode commonly used in critically ill patients (Esteban et al., 2013). The maintenance of spontaneous respiratory activity in acute respiratory distress syndrome (ARDS) patients improves respiratory function and decreases the need for vasopressor and sedative drugs (Putensen et al., 2001). Assisted ventilation modes have been commonly used in the management of patients with ARDS, in particular those with mild to moderate hypoxemic respiratory failure (Bellani et al., 2016).

In the last years, researchers have proposed to vary the level of pressure support on a breath-by-breath basis to restore the physiological variability of breathing activity (Tobin et al., 1988). Variable pressure support ventilation (vPSV), compared with conventional PSV, improved oxygenation in the experimental models of ARDS (Gama de Abreu et al., 2008) and ventilator-patient synchrony in a small pilot study in critically ill patients with acute respiratory failure (Spieth et al., 2013). These effects could be mediated by an amelioration of the ventilation-perfusion matching (Huhle et al., 2016), as well as a recruitment effect due to the repetitive delivery of breaths with a higher tidal volume, which might also result in a reduction of lung inhomogeneity (Mauri et al., 2017). However, so far, the only clinical study published has used only one variability level at fixed pressure support (Δ*P*_S_) (Spieth et al., 2013). Therefore, the effects of different levels of variability and the impact of variability at different Δ*P*_S_ levels remain unknown. Different levels of variability might modify differently the ventilation perfusion-matching and might affect differently gas exchange and respiratory mechanics.

The aim of this study was to evaluate the effects of vPSV, at different levels of variability and pressure support, on short-term lung function parameters in patients with mild to moderate ARDS. We tested the hypothesis that vPSV would improve gas exchange, respiratory mechanics, and patient-ventilator asynchrony. We also hypothesized that the degree of variability and the level of Δ*P*_S_ would influence the effects of vPSV.

## Methods

### Study Design

This was a prospective, crossover, randomized clinical trial conducted in a single university hospital intensive care unit (ICU).

### Inclusion and Exclusion Criteria

Patients aged >18 years with mild to moderate ARDS (PaO_2_/FIO_2_ ratio between 100 and 300 mmHg with a positive end-expiratory pressure, PEEP ≥5 cmH_2_O) already receiving PSV per clinical indication were screened for inclusion. Exclusion criteria were pregnancy, chronic obstructive pulmonary disease, presence of pneumothorax or chest tubes, and unavailability of research staff.

### Interventions

**Figure 1:**
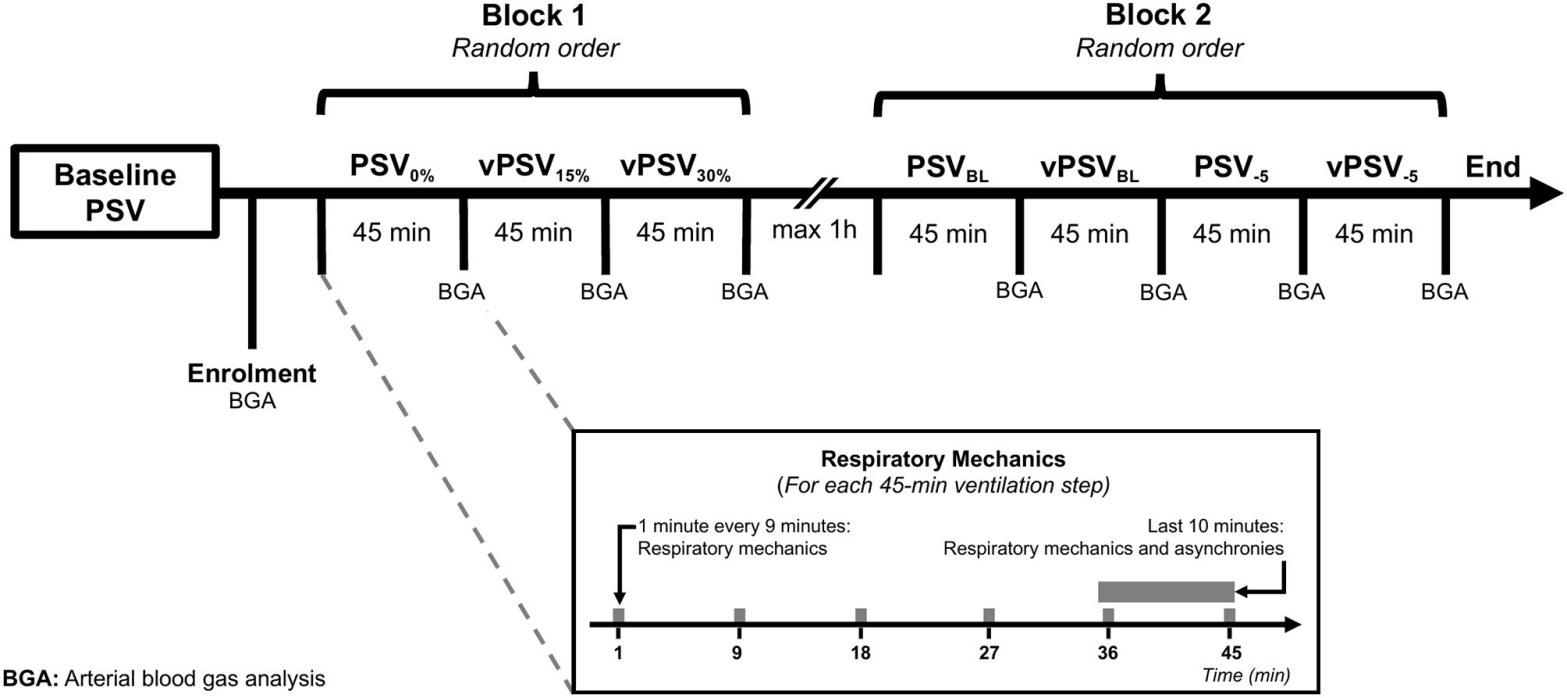
Time course of interventions. Within each intervention block, different ventilation settings were delivered in random order. PSV_0%_, conventional PSV ventilation with no variability; vPSV_15%_, variable PSV with variability set to 15% CV; vPSV_30%_, variable PSV with variability set to 30% CV; PSV_BL_, PSV with no variability and baseline Δ*P*_S_ as per clinical indication; vPSV_BL_, variable pressure support with variability set to achieve ±5 cmH_2_O and baseline Δ*P*_S_ as per clinical indication; PSV_−5_, PSV with no variability and Δ*P*_S_ reduced by 5 cmH_2_O from the baseline value; vPSV_−5_, variable PSV with variability set to achieve ±5 cmH_2_O and Δ*P*_S_ reduced by 5 cmH_2_O from the baseline value; PSV, pressure support ventilation; CV, coefficient of variation.

According to the local clinical practice, conventional PSV was delivered by an Evita Infinity V500 ventilator (Dräger Medical AG, Lübeck, Germany) targeting a *V*_T_ of 6–8 ml/kg of predicted body weight, respiratory rate ≤ 25 min^−1^ with PEEP and FIO_2_ titrated to achieve a peripheral oxygen saturation ≥92%. This ventilator can operate in vPSV mode setting the variability of the Δ*P*_S_ and delivers breaths with an approximately Gaussian distribution, truncated at 3 SDs from the mean Δ*P*_S_. The parameter “variability” of this ventilator refers to the range of Δ*P*_S_, e.g., 90% “variability” results in a 30% coefficient of variation (CV). As illustrated in Figure 1, all patients underwent two blocks of interventions, receiving 45-min periods of ventilation with different settings. In the first block, the effects of three levels of variability were tested at constant Δ*P*_S_ to explore the effect of variability added to a fixed Δ*P*_S_ level, while in the second block, a variability of ±5 cmH_2_O was added to Δ*P*_S_ set at either the baseline level or the baseline level minus 5 cmH_2_O, to investigate the effects of variability at two Δ*P*_S_ levels. During the first block, the Δ*P*_S_ was set at a fixed value corresponding to the level chosen by the treating clinician before enrolment, and three different CV% levels were used: 0% (PSV_0%_), 15% (vPSV_15%_), and 30% (vPSV_30%_). During the second block, four ventilation settings were used: PSV with baseline Δ*P*_S_ (PSV_BL_), baseline Δ*P*_S_ with variability set individually to ±5 cmH_2_O (vPSV_BL_), Δ*P*_S_ reduced by 5 cmH_2_O compared with the baseline with either no variability (PSV_−5_) or variability set to ±5 cmH_2_O (vPSV_−5_). The two blocks were performed sequentially, within 1 h from each other to allow for nursing assistance if required, and ventilation modes within each intervention block were assigned in random order with a Latin square design (as shown in Figure 1; Supplementary Figures 1, 2). The randomization sequence was generated with an online service, and a sealed envelope was opened at the moment of patient enrolment. Participants were blinded to the treatment assignment as were the operators involved in respiratory mechanics analysis.

Patient management procedures not related to mechanical ventilation, including sedation and fluid administration, were at the discretion of the treating clinician. When clinically feasible, we avoided changing FIO_2_, PEEP, and Δ*P*_S_ during the study, and in case of desaturation below 92%, FIO_2_ increase was prioritized over PEEP increase. After completion of the study protocol, ventilation was continued at the discretion of the treating physician.

### Measurements

An esophageal balloon catheter (Compliance catheter, Microtek Medical B.V., Zutphen, The Netherlands) was inserted through the nose or mouth, filled with 1.5 ml, and correct positioning was verified with an occlusion maneuver (Akoumianaki et al., 2014). The flow was measured with a heated Fleisch-type pneumotachograph connected to a multi-channel transducer (ICU Lab, KleisTEK Engineering, Bari, Italy), while the tidal volume was measured as the integral of flow over time. Respiratory traces were recorded continuously throughout the study. An arterial blood gas analysis, heart rate, and invasive mean arterial pressure were recorded at baseline and the end of each ventilation step.

Pressure-time and flow-time curves were analyzed offline with a dedicated script written in MATLAB (MathWorks, MA, USA). The following parameters were computed breath by breath: V_T_, PEEP, Δ*P*_S_, mean airway pressure, inspiratory time to total time ratio (*T*_insp_/*T*_tot_), respiratory rate (RR), esophageal pressure swings (Δ*P*_es_), and peak transpulmonary pressure (P_L_). The respiratory muscle activity was quantified with the esophageal pressure-time product per min (PTP_es_), calculated as follows (Mauri et al., 2016):


$$\begin{array}{l} PTP_{\;es,min}=RR\cdot \int P_{mus}\;dt=RR\cdot \int (P_{cw,recoil}-P_{es})\;dt \end{array}$$

where *P*_mus_ is the pressure generated by the respiratory muscles, and *P*_cw,recoil_ is the chest wall recoil pressure, calculated assuming a fixed elastance of 5 cmH_2_O/L. The asynchrony index was computed as the number of asynchronous events divided by the total number of ventilator cycles plus ineffective efforts during expiration multiplied by 100 (Blanch et al., 2015). Asynchronies were classified independently by two experienced operators (LB and MV), and discrepancies were resolved by consensus. The analysis of respiratory mechanics data was performed by three operators blinded to the ventilation settings (ADO, MF, and LM). Also, we measured the evolution of respiratory mechanics at min 1, 9, 18, 27, 36, and 45 from the start of each ventilation step. To allow sufficient time for patient adaptation, main analyses of respiratory mechanics and asynchronies were restricted to the last 10 min of each ventilation step.

### Data Analysis and Sample Size Calculation

All variables are reported as medians [25th−75th percentile], if not otherwise specified. Measurements on multiple breaths were aggregated within-patients computing the median and the CV; then, between-patients medians [25th−75th percentile] were computed. Comparisons between continuous variables during the different ventilation steps were sought with Friedman's test and Dunn's *post-hoc* test. The primary endpoint was the partial pressure of arterial oxygen to FiO_2_ ratio (PaO_2_/FiO_2_). From internal administrative data, we expected a baseline PaO_2_/FiO_2_ around 150 ± 50 mmHg. Using a Latin square crossover design, and assuming an intra-subject correlation of the PaO_2_/FiO_2_ between treatments with ρ = 0.75, we needed to enroll at least 16 patients to achieve 90% power (1-β) to detect a 20% relative increase in the PaO_2_/FiO_2_ ratio (Muller and Barton, 1989; Muller et al., 1992). To account for potential drop-off or missing respiratory mechanics data, we aimed to enroll 20 patients. Repeated measurement analysis of respiratory mechanics parameters at different timepoints within each ventilation block was performed using mixed-effects linear models using patients as random effects and timepoint, ventilation, and their interaction as fixed effects.

In one *post-hoc* analysis, associations were determined between the respiratory mechanics parameters of each breath and the Δ*P*_S_ received during the preceding breath in the vPSV_BL_ and vPSV_−5_ ventilation steps. For this purpose, mixed-effects linear models were used, using patients as random effects and the Δ*P*_S_ received during the preceding breath as the fixed effect.

All analyses were performed with R 3.2.3 (The R Foundation for Statistical Computing, www.r-project.org). Statistical significance was considered for two-tailed *p* < 0.05.

## Results

**Figure 2:**
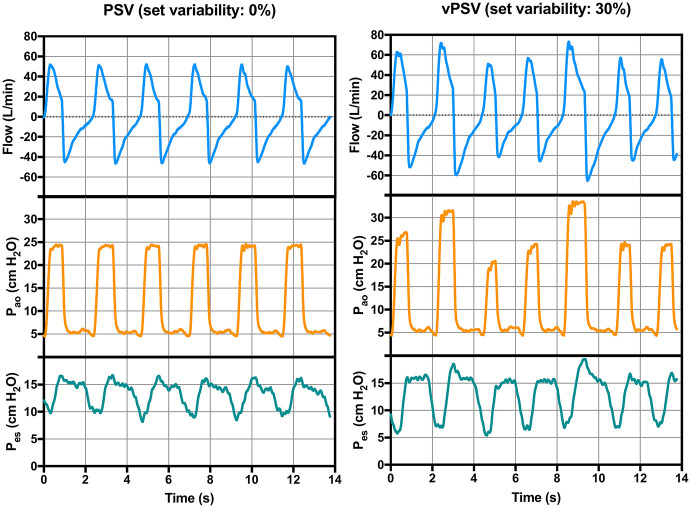
Representative respiratory traces of a patient during conventional (left) and variable (right) pressure support ventilation. *P*_ao_, pressure at the airway opening; *P*_es_, esophageal pressure.

**Figure 3:**
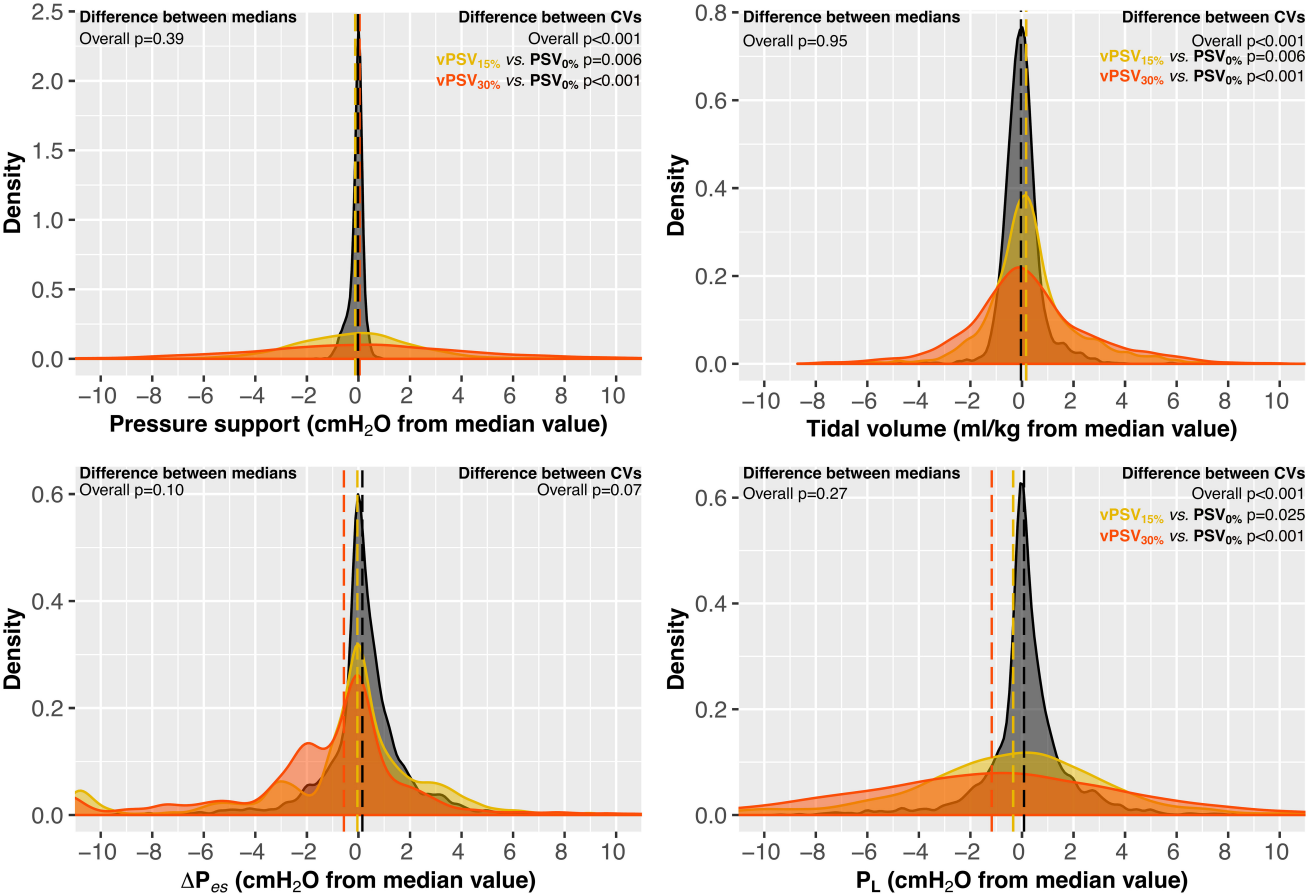
Respiratory mechanics at different levels of variability (block 1). Variables are reported as the difference from the median value achieved during PSV_0%_ to allow between-patients visual comparisons. Dashed lines represent the medians of each ventilation step. PSV_0%_, conventional PSV ventilation with no variability; vPSV_15%_, variable PSV with variability set to 15% CV; vPSV_30%_, variable PSV with variability set to 30% CV; CV, coefficient of variation; Δ*P*_S_, pressure support; Δ*P*_es_, esophageal pressure swings; *P*_L_, peak transpulmonary pressure.

Twenty patients were enrolled and completed the study. Baseline characteristics are presented in Table 1. The FiO_2_ and PEEP were kept constant during the study in all patients; one patient required Δ*P*_S_ reduction between ventilation block 1 and block 2 according to the treating clinician decision for reasons unrelated to the study procedures. Tables 2, 3 show respiratory mechanics, hemodynamics, and arterial blood gas analysis in ventilation blocks 1 and 2, respectively. The distribution of key respiratory mechanics parameters in ventilation blocks 1 and 2 is illustrated in Figures 2, 3, respectively. Supplementary Figures 3–11 report details the evolution over time of the respiratory mechanics parameters in the different ventilation steps.

**Table 1:** Baseline characteristics of patients.

**Patient characteristics**	
Number of patients	20
Age (years)	72 [59–79]
Female sex (N, %)	6/20 (30%)
Weight (kg)	80 [64–87]
Height (cm)	175 [165–180]
Body mass index (kg/m^2^)	25.8 [22.7–29.5]
PBW (kg)	71 [58–75]
SAPS II	53 [38–60]
SOFA	7 [6–9]
RASS	−3 [−3 to −1]
Sedative drugs (N, %)	Propofol 5/20 (25%) Dexmedetomidine 3/20 (15%) Midazolam 4/20 (20%) None 8/20 (40%)
Analgesic drugs (N, %)	Fentanyl 8/20 (40%) Morphine 2/20 (10%) None 10/20 (50%)
Days of ventilation prior to inclusion	7 [5–9]
Primary reason for admission to the ICU	Acute respiratory failure: 10 (50%) Multiple trauma: 4 (20%) Brain hemorrhage: 3 (15%) Post-cardiac arrest: 3 (15%)
Risk factor for development of ARDS	Pneumonia: 11 (55%) Multiple fractures: 2 (10%) Sepsis: 4 (20%) Aspiration pneumonia: 3 (15%)
Blood gas analysis at enrolment
PaO_2_ (mmHg)	97 [79–120]
PaCO_2_ (mmHg)	40 [36–46]
pHa	7.46 [7.44–7.51]
PaO_2_/FIO_2_ ratio (mmHg)	198 [154–250]
Ventilator settings at enrolment	
Δ*P*_S_ (cmH_2_O)	15 [14–17]
PEEP (cmH_2_O)	6 [5–8]
FIO_2_ (%)	50 [43–58]
Tidal volume (mL/kg of PBW)	7.5 [7.0–8.5]
Respiratory rate (min^−1^)	15 [13–20]

*PBW, predicted body weight; PEEP, positive end-expiratory pressure; ΔP_S_, pressure support; SAPS, simplified acute physiology score; SOFA, sequential organ failure assessment score; RASS, richmond agitation-sedation scale; ICU, intensive care unit*.

**Table 2:** Gas exchange, hemodynamics, and respiratory mechanics in patients during pressure support ventilation at different levels of variability (Block 1).

	**Ventilation modes**			***p*** **-values**		
	**PSV_**0%**_**	**vPSV_**15%**_**	**vPSV_**30%**_**	**Overall**	**vPSV_**15%**_ vs. PSV_**0%**_**	**vPSV_**30%**_ vs. PSV_**0%**_**
**Ventilation settings**						
Δ*P*_S,set_ (cmH_2_O)	15.0 [13.0–16.0]	15.0 [13.0–16.0]	15.0 [13.0–16.0]	>0.99		
Δ*P*_S_ set variability (CV %)	0	15	30	<0.001		
**Gas exchange**						
PaO_2_/FIO_2_ (mmHg)	209 [157–242]	214 [160–256]	210 [179–252]	0.62		
PaO_2_ (mmHg)	96 [79–118]	98 [85–116]	108 [82–123]	0.62		
PaCO_2_ (mmHg)	44 [37–46]	43 [38–46]	43 [37–48]	0.95		
pH	7.45 [7.43–7.49]	7.49 [7.43–7.50]	7.47 [7.44–7.50]	0.64		
**Hemodynamics**						
Heart rate (min^−1^)	85 [72–92]	83 [75–90]	83 [73–91]	0.46		
Mean arterial pressure (mmHg)	78 [68–96]	79 [73–91]	82 [70–90]	0.37		
**Respiratory mechanics**						
Δ*P*_S,measured_ (cmH_2_O)	14.6 [12.5–15.7]	14.6 [12.7–16.0]	14.7 [12.6–16.5]	0.39		
PS_measured_ (CV, %)	1.2 [0.7–2.0]	15.6 [15.0–17.2]^a^	29.7 [27.5–31.5]^a^	<0.001	0.006	<0.001
Total PEEP (cmH_2_O)	7.2 [6.1–8.5]	7.4 [6.2–8.6]	7.5 [6.2–8.4]	0.09		
Total PEEP (CV, %)	2.2 [1.1–3.1]	2.2 [1.2–3.8]	2.6 [1.7–3.3]^a^	0.034	0.40	0.026
*P*_mean_ (cmH_2_O)	11.6 [9.9–12.6]	12.1 [10.1–12.9]	11.6 [10.4–12.8]	0.16		
*P*_mean_ (CV, %)	3.7 [3.0–6.0]	8.1 [5.8–9.6]^a^	13.2 [10.2–15.5]^a^	<0.001	0.016	<0.001
Respiratory rate (min^−1^)	16.7 [13.7–21.4]	16.8 [13.9–21.4]	15.6 [13.8–19.7]	0.86		
Respiratory rate (CV, %)	11.6 [9.2–15.8]	12.5 [10.3–22.4]	17.9 [15.8–24.9]^a^	0.002	0.17	<0.001
*V*_T_ (ml/kg of PBW)	8.1 [7.3–10.0]	8.8 [7.0–10.7]	8.9 [7.2–10.1]	0.95		
*V*_T_ (CV, %)	6.7 [4.5–9.1]	13.1 [10.7–14.4]^a^	23.8 [17.8–28.1]^a^	<0.001	0.006	<0.001
*T*_insp_/*T*_tot_	0.34 [0.29–0.41]	0.37 [0.32–0.41]	0.37 [0.30–0.43]	0.35		
*T*_insp_/*T*_tot_ (CV, %)	11.1 [7.4–15.1]	10.9 [9.6–16.3]	14.6 [12.3–21.0]	0.08		
PTP_es_ (cmH_2_O s min ^−1^)	126 [102–226]	154 [103–194]	136 [121–208]	0.95		
PTP_es_ (CV, %)	26.8 [15.6–39.2]	30.1 [17.0–47.6]	36.2 [25.9–58.2]^a^	0.029	0.71	0.026
Δ*P*_es_ (cmH_2_O)	5.0 [2.1–7.6]	3.0 [1.3–7.4]	2.7 [1.6–5.4]	0.10		
Δ*P*_es_ (CV, %)	23.2 [18.0–34.2]	26.8 [19.7–40.9]	26.8 [24.3–47.5]	0.07		
*P*_L_ (cmH_2_O)	18.0 [16.9–21.4]	17.8 [16.0–21.9]	17.4 [15.9–20.2]	0.27		
*P*_L_ (CV, %)	4.5 [2.7–11.7]	14.7 [13.2–15.9]^a^	25.8 [21.4–27.3]^a^	<0.001	0.025	<0.001
Asynchrony index (%)	1.6 [0.6–10.5]	2.2 [0.5–16.3]	5.1 [1.0–17.4]^a^	0.031	0.21	0.019

*Values are computed during the last 10 min of a 45-min ventilation period. Data are reported as inter-subject median [25th−75th percentile] of the intra-subject median values*.

a *Significantly different from PSV_0%_. PSV_0%_, pressure support ventilation with no variability; vPSV_15%_, variable pressure support ventilation with 15% CV variability; vPSV_30%_, variable pressure support ventilation with 30% CV variability*.

*CV, coefficient of variation; PEEP, positive end-expiratory pressure; PBW, predicted body weight; ΔP_S_, pressure support; V_T_, tidal volume; PTP, esophageal pressure-time product; ΔP_es_, esophageal pressure swings; P_L_, peak transpulmonary pressure*.

**Table 3:** Gas exchange, hemodynamics, and respiratory mechanics in patients during pressure support ventilation at different variability and pressure support level (Block 2).

	**Ventilation modes**				***p*** **-values**			
	**PSV_**BL**_**	**vPSV_**BL**_**	**PSV_**−5**_**	**vPSV_**−5**_**	**Overall**	**vPSV_**BL**_ vs. PSV_**BL**_**	**PSV_**−5**_ vs. PSV_**BL**_**	**vPSV_**−5**_ vs. PSV_**−5**_**
**Ventilator Settings**								
Δ*P*_S_ setting	Baseline		Baseline−5 cmH_2_O					
Δ*P*_S,set_ (cmH_2_O)	14.0 [12.0–16.0]	14.0 [12.0–16.0]	9.0 [7.0–11.0]	9.0 [7.0–11.0]	<0.001	0.99	<0.001	<0.001
Variability setting	None	± 5 cmH_2_O	No variability	± 5 cmH_2_O				
Δ*P*_S_ set variability (CV %)	0 [0–0]	11 [9–13]	0 [0–0]	15 [13–20]	<0.001	<0.001	0.99	<0.001
**Gas exchange**								
PaO_2_/FIO_2_ (mmHg)	213 [180–229]	194 [180–229]	215 [183–239]	197 [167–224]	0.61			
PaO_2_ (mmHg)	95 [89–117]	99 [87–115]	99 [90–121]	98.8 [85–1112]	0.61			
PaCO_2_ (mmHg)	42 [39–46]	43 [39–48]	44 [40–49]	44 [40–51]	0.18			
pH	7.47 [7.43–7.49]	7.48 [7.43–7.49]	7.47 [7.43–7.48]	7.47 [7.42–7.48]	0.21			
**Hemodynamics**								
Heart rate (min^−1^)	83 [77–93]	84 [77–92]	84 [77–92]	86 [76–92]	0.46			
Mean arterial pressure (mmHg)	88 [81–93]	82 [78–87]	80 [77–90]	86 [77–90]	0.37			
Respiratory mechanics								
Δ*P*_S,measured_ (cmH_2_O)	13.2 [12.0–15.8]	13.6 [12.5–15.9]	8.4 [7.0–10.5]^a^	8.5 [7.2–10.7]	<0.001	0.92	<0.001	0.67
Δ*P*_Smeasured_ (CV, %)	1.5 [0.9–4.8]	12.1 [11.1–14.3]^a^	2.3 [1.7–6.6]	17.9 [15.2–18.6]^b^	<0.001	0.001	0.99	<0.001
Total PEEP (cmH_2_O)	7.6 [6.2–8.4]	7.5 [6.2–8.5]	7.7 [5.8–8.7]^a^	7.7 [5.7–8.7]	0.001	0.99	0.009	0.92
Total PEEP (CV, %)	2.3 [1.4–4.9]	2.6 [1.8–8.0]	2.1 [1.6–4.3]	2.4 [1.4–5.9]	0.09			
*P*_mean_ (cmH_2_O)	11.0 [9.7–12.6]	11.2 [9.7–13.1]	9.7 [8.4–11.9]^a^	9.8 [8.4–11.8]	<0.001	0.67	<0.001	0.99
*P*_mean_ (CV, %)	5.4 [3.4–8.2]	8.1 [5.5–10.2]^a^	2.5 [1.8–5.0]	6.0 [4.5–7.3]^b^	<0.001	0.014	0.11	0.029
Respiratory rate (min ^−1^)	14.7 [13.7–18.3]	17.6 [14.8–19.4]	22.9 [16.0–24.9]^a^	20.1 [16.9–26.8]	0.022	0.96	0.041	0.95
Respiratory rate (CV, %)	18.8 [10.6–46.4]	32.0 [16.1–60.3]	13.1 [6.2–35.2]	14.6 [9.7–17.7]	0.003	0.43	0.43	0.99
*V*_T_ (ml/kg of PBW)	8.5 [7.2–9.4]	8.2 [7.0–9.1]	7.0 [5.9–7.6]^a^	7.2 [6.0–7.7]	<0.001	0.92	<0.001	0.67
*V*_T_ (CV, %)	9.3 [5.1–15.7]	12.7 [11.1–15.3]^a^	8.3 [4.2–13.6]	10.8 [9.4–14.6]	0.003	0.006	0.99	0.74
*T*_insp_/*T*_tot_	0.36 [0.30–0.37]	0.37 [0.30–0.39]	0.36 [0.32–0.39]	0.35 [0.32–0.38]	0.42			
*T*_insp_/*T*_tot_ (CV, %)	9.3 [5.1–15.7]	12.7 [11.1–15.3]^a^	8.3 [4.2–13.6]	10.8 [9.4–14.6]	0.058			
PTP_es_ (cmH_2_O s min^−1^)	155.2 [118.4–262.8]	161.4 [87.1–248.3]	215.0 [128.1–357.9]	259.1 [151.1–422.8]	0.001	0.51	0.18	0.99
PTP_es_ (CV, %)	31.8 [20.6–52.3]	53.8 [23.9–71.1]	27.5 [16.0–40.5]	30.7 [18.5–44.7]	0.005	0.08	0.75	0.81
Δ*P*_es_ (cmH_2_O)	4.4 [2.1–9.3]	5.3 [1.3–8.0]	5.6 [1.6–11.8]	10.4 [2.6–14.1]	<0.001	0.59	0.59	0.43
Δ*P*_es_ (CV, %)	32.7 [21.1–45.5]	40.9 [22.3–53.5]	22.8 [13.1–31.5]	20.6 [12.7–32.2]	<0.001	0.51	0.29	0.67
*P*_L_ (cmH_2_O)	18.5 [15.9–23.2]	18.6 [16.1–23.4]	14.6 [11.5–20.8]^a^	18.0 [14.0–21.6]	<0.001	0.99	<0.001	0.67
*P*_L_ (CV, %)	7.5 [3.8–12.6]	12.2 [11.1–17.0]^a^	10.0 [4.2–13.1]	15.7 [12.4–17.3]	0.009	0.042	0.95	0.14
Asynchrony index (%)	1.5 [0.7–7.2]	2.4 [0.1–12.6]	0.9 [0.0–7.6]	1.3 [0–3.9]	0.21			

*Values are computed during the last 10 min of a 45-min ventilation period. Data are reported as inter-subject median [25th−75th percentile] of the intra-subject median values*.

a *Significant difference compared to PSV_BL_ (p < 0.05)*.

b *Significant difference compared to PSV_−5_ (p < 0.05)*.

*PSV_BL_, pressure support ventilation with no variability and baseline ΔP_S_ as per clinical indication; vPSV_BL_, variable pressure support with variability set to achieve ±5 cmH_2_O and baseline ΔP_S_ as per clinical indication; PSV_−5_, pressure support ventilation with no variability and ΔP_S_ reduced by 5 cmH_2_O from the baseline value; vPSV_−5_, variable pressure support ventilation with variability set to achieve ±5 cmH_2_O and ΔP_S_ reduced by 5 cmH_2_O from the baseline value; CV, coefficient of variation; PEEP, positive end-expiratory pressure; PBW, predicted body weight; ΔP_S_, pressure support; V_T_, tidal volume; PTP, esophageal pressure-time product; ΔP_es_, esophageal pressure swings; P_L_, peak transpulmonary pressure*.

### Block 1: Physiological Effects of Different Variability Levels at Constant ΔP_S_

The PaO_2_/FiO_2_ did not differ between ventilation steps in block 1 (*p* = 0.62, Table 2). Median respiratory mechanics variables, other gas exchange, and hemodynamic parameters did not change between vPSV_15%_ and vPSV_30%_ compared with PSV_0%_ (Table 2). However, the variability of Δ*P*_S_, PEEP_tot_, *P*_mean_, and *V*_T_ was higher with PSV_15%_ and PSV_30%_ compared with PSV_0%_ (Table 2). The RR and PTP_es,min_ had higher variability only with vPSV_30%_ (Table 2). Moreover, asynchronies were more frequent with vPSV_30%_ compared with PSV_0%_ (*p* = 0.019, Table 2).

### Block 2: Physiological Effects of Variability at Two Levels of ΔP_S_

The PaO_2_/FiO_2_, as well as other gas exchange and hemodynamic parameters, did not differ between ventilation steps in block 2 (Table 3). Ventilation modes with Δ*P*_S_ reduced by 5 cmH_2_O (PSV_−5_ and vPSV_−5_) had lower *P*_mean_, *V*_T_, *P*_L_, and higher RR (Table 3). Adding ±5 cmH_2_O variability (vPSV_BL_ and vPSV_−5_ steps) increased the variability of Δ*P*_S_ and *P*_mean_ compared to PSV without variability at the corresponding Δ*P*_S_ level. Adding ±5 cmH_2_O variability increased the variability of *V*_T_ and *P*_L_ only when using the baseline Δ*P*_S_, but not when the Δ*P*_S_ was reduced by 5 cmH_2_O. The incidence of asynchronies was not different between ventilation steps in block 2 (Table 3).

Tables 2, 3 report extensive details on respiratory mechanics, hemodynamics, and arterial blood gas analysis in ventilation blocks 1 and 2, respectively. The distribution of key respiratory mechanics parameters in ventilation blocks 1 and 2 is illustrated in Figures 3, 4, respectively. Supplemental Figures 3–11 report details the evolution over time of the respiratory mechanics parameters in the different ventilation steps.

**Figure 4:**
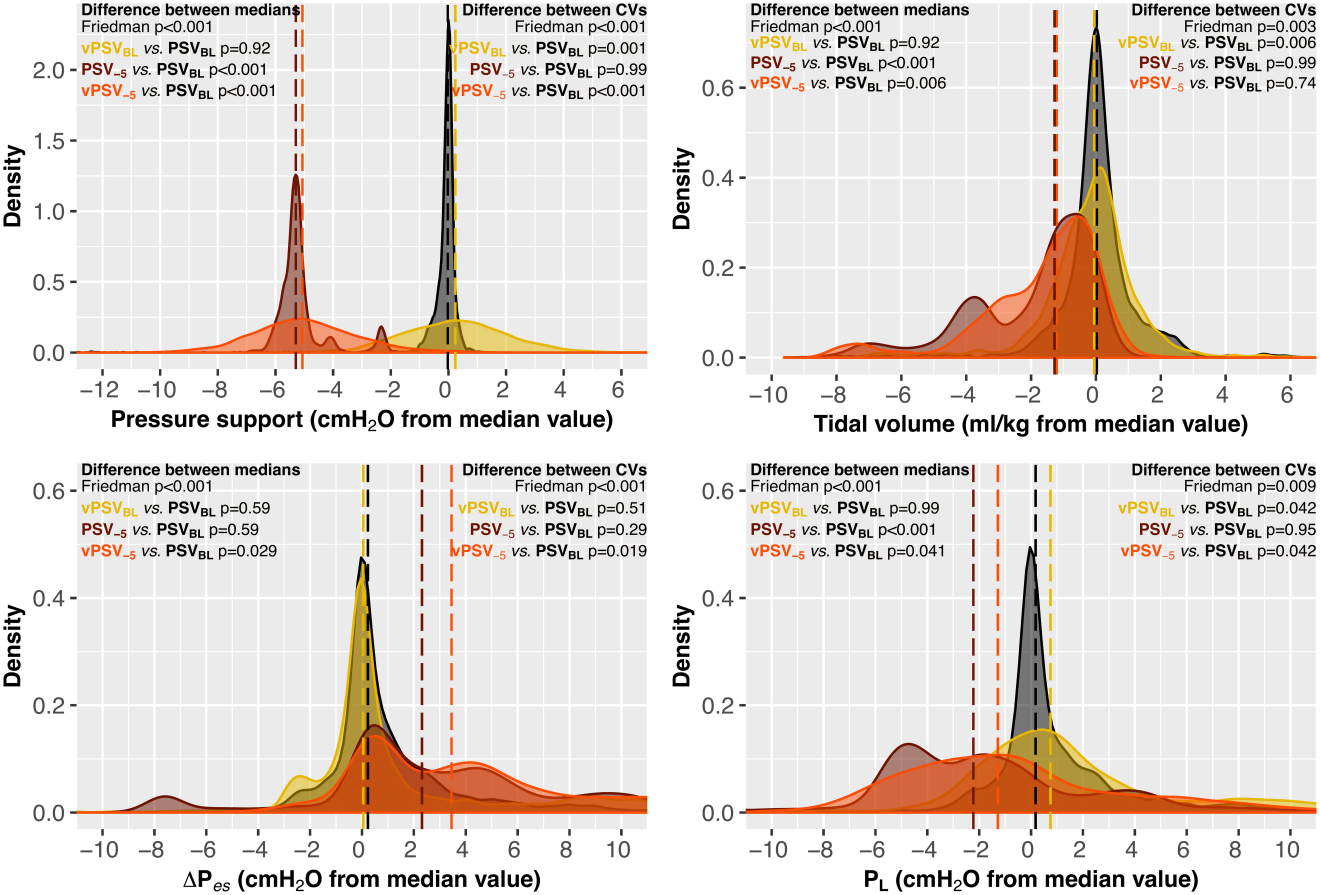
Effects of variability on the distribution of respiratory mechanics parameters at different levels of pressure support (block 2). Variables are reported as the difference from the median value achieved during PSV_BL_ to allow between-patients visual comparisons. Dashed lines represent the medians of each ventilation step. PSV_BL_, PSV with no variability and baseline Δ*P*_S_ as per clinical indication; vPSV_BL_, variable pressure support with variability set to achieve ±5 cmH_2_O and baseline Δ*P*_S_ as per clinical indication; PSV_−5_, PSV with no variability and Δ*P*_S_ reduced by 5 cmH_2_O from the baseline value; vPSV_−5_, variable PSV with variability set to achieve ±5 cmH_2_O and Δ*P*_S_ reduced by 5 cmH_2_O from the baseline value; PSV, pressure support ventilation; CV, coefficient of variation; Δ*P*_S_, pressure support; Δ*P*_es_, esophageal pressure swings; *P*_L_, peak transpulmonary pressure.

### *Post-hoc* Analysis

**Figure 5:**
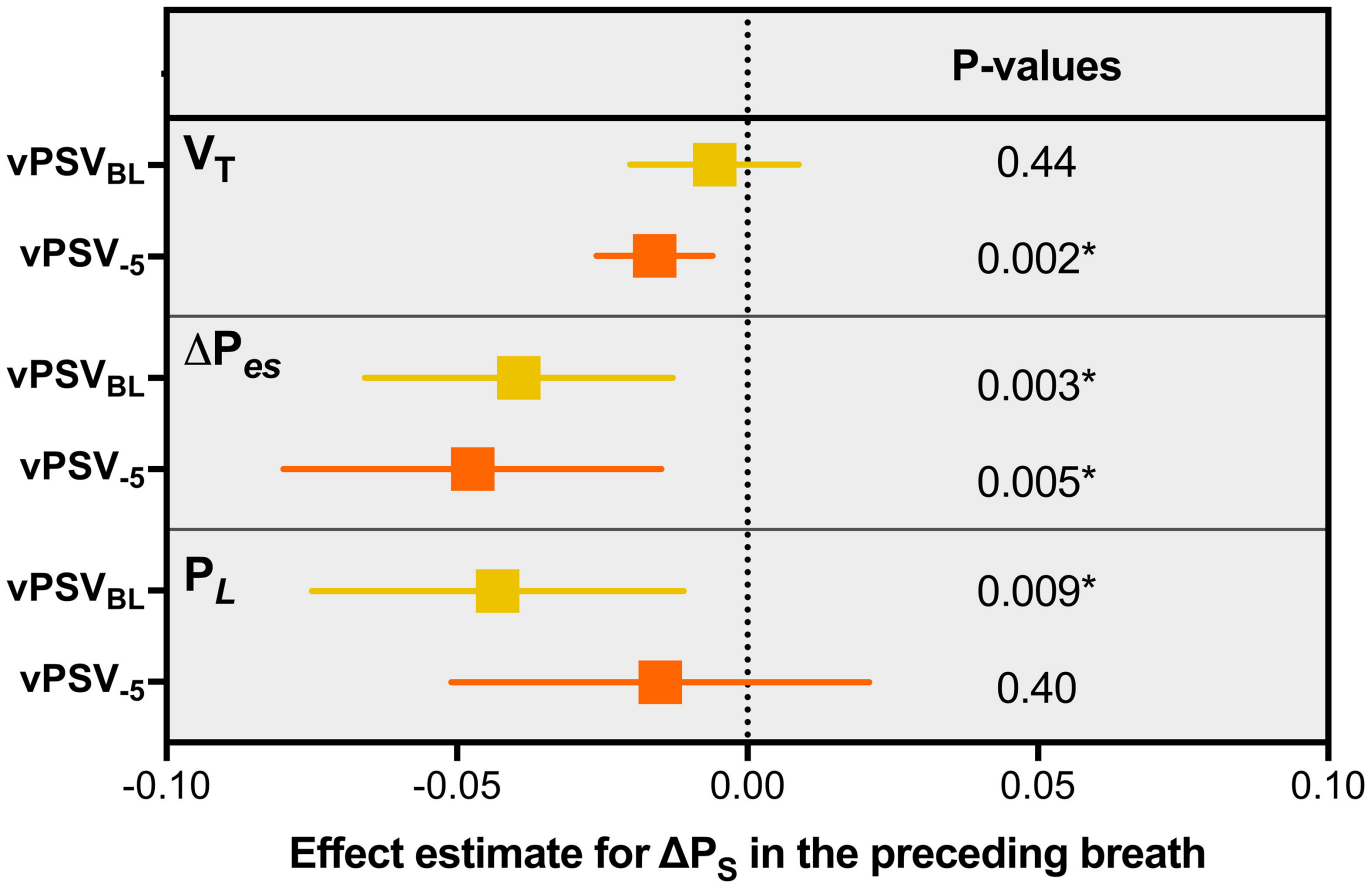
Associations between respiratory mechanics parameters and the pressure level received in the preceding breath during variable PSV. Squares and confidence intervals refer to the effect estimate for Δ*P*_S_ in a mixed model comprising the Δ*P*_S_ received during the preceding breath as a fixed effect and the patient as a random effect with random intercept. The units of the estimates are expressed in the untransformed units of the variables, i.e., they represent the absolute change in *V*_T_, Δ*P*_es_, or *P*_L_ when the Δ*P*_S_ received during the preceding breath increases by 1 cmH_2_O. vPSV_BL_, variable PSV with variability set to achieve ±5 cmH_2_O and baseline Δ*P*_S_ as per clinical indication; vPSV_−5_, variable PSV ventilation with variability set to achieve ±5 cmH_2_O and Δ*P*_S_ reduced by 5 cmH_2_O from the baseline value; PBW, predicted body weight; PSV, pressure support ventilation; Δ*P*_S_, pressure support; *V*_T_, tidal volume; Δ*P*_es_, esophageal pressure swings; *P*_L_, peak transpulmonary pressure. ^*^Significant association (*p* < 0.05).

Associations between respiratory mechanics parameters and the pressure level received in the preceding breath during vPSV_BL_ and vPSV_−5_ are reported in Figure 5. The Δ*P*_S_ received in the preceding breath was inversely associated with the magnitude of the inspiratory effort (Δ*P*_es_) in the following breath, both during vPSV_BL_ (*p* = 0.003) and vPSV_−5_ (*p* = 0.005).

## Discussion

The main findings of this study are that in our mixed-ICU population of patients with mild to moderate ARDS: (1) vPSV with 15 or 30% variability did not influence gas exchange compared with conventional PSV; (2) at constant Δ*P*_S_, vPSV increased the variability of *V*_T_ and *P*_L_; (3) vPSV_30%_ increased the incidence of asynchronies; and (4) when the Δ*P*_S_ was reduced by 5 cmH_2_O from the baseline value, adding variability did not increase the variability of *V*_T_ and *P*_L_.

This is the first study comparing the short-term effects of vPSV at different levels of variability and Δ*P*_S_ in patients with ARDS. In previous studies, vPSV improved oxygenation in the experimental models of ARDS (Gama de Abreu et al., 2008; Spieth et al., 2011, 2012), but not in a cohort of hypoxemic critically ill patients (Spieth et al., 2013). However, that last study included mostly postoperative patients without a confirmed diagnosis of ARDS and investigated a single level of variability and pressure support. Opposite to what was found in preclinical studies in animals, vPSV had no effect on gas exchange, when the Δ*P*_S_ was set to the baseline value identified by the treating clinician and neither when it was reduced by 5 cmH_2_O. This could be explained by several mechanisms; most importantly, the time investigated in each ventilation step was relatively short, and the fact that patients had an established diagnosis of ARDS mostly in their recovery phase and received mechanical ventilation for few days prior to the inclusion in this study. Under these conditions, patient lungs could have developed consolidation, namely, the presence of lung regions scarcely responsive to recruitment (Cressoni et al., 2017). In this case, the breaths with higher Δ*P*_S_ received cyclically during variable pressure support might expose the patient to volutrauma in the aerated regions of the lung (Güldner et al., 2016; Pelosi et al., 2016) due to the reduced size of the lung aerated compartment. Another explanation for the possible lack of effect of variability on oxygenation might be that, different from what happens in PSV with a sigh, vPSV has no control over the time spent at higher pressure during tidal breathing. This might result in random breaths with higher *P*_S_ and short inspiratory time, both possibly insufficient to achieve recruitment. The tidal volume measured in this cohort was higher than the recommended targets, but this reflects the current clinical practice in patients with ARDS receiving assisted ventilation modes (Bellani et al., 2016; Writing Group for the PReVENT Investigators et al., 2018). During the second block of ventilations, the patients tolerated a Δ*P*_S_ reduction without worsening the gas exchange in the short term, at the price of a modest increase of the respiratory rate, suggesting that they were slightly over-assisted. This could have influenced patient-ventilator interaction (Kataoka et al., 2018) and the response to variability, as suggested by the finding that, during the second block of interventions, the variability of *V*_T_ was increased by vPSV compared with PSV only when the baseline Δ*P*_S_ was used. However, during ventilation steps with baseline Δ*P*_S_, patients had a work of breathing estimated with the PTP_es_ of around 150 cmH_2_O·s·min^−1^, which is within the recommended range (Mauri et al., 2016). Interestingly, higher *P*_S_ resulted in a reduction in Δ*P*_ES_ in the following breath at both set Δ*P*_S_ levels, while the variability of *V*_T_ and *P*_L_ was increased by extrinsic variability only at higher Δ*P*_S_. This seems to suggest that while a neural response to extrinsic variability is present independent of the level of assistance, its effects on the variability of *V*_T_ and *P*_L_ are influenced by the level of Δ*P*_S_.

This study is underpowered to demonstrate the effects of vPSV on patient-centered outcomes like duration of ventilation. This is tested in another, yet ongoing clinical trial (Kiss et al., 2013). In the *post-hoc* analysis, the effects of vPSV on the response of patients in terms of inspiratory effort, transpulmonary pressure, and tidal volume developed in the following breath were studied. An inverse association between the Δ*P*_S_ received in the preceding breath and the inspiratory effort was observed. Different from other modified PSV modes such as the proportional assist ventilation (PAV) and the neurally adjusted ventilatory assist (NAVA), the variability of Δ*P*_S_ was random, i.e., is not related to the efforts of patients. This analysis suggests that there might be a complex interaction between the ventilator and a patient, in which the inspiratory effort and the adaptation of the patient to pressure support are influenced by the history of the previous breaths.

### Limitations

This study has several limitations. The crossover design allowed the investigation of the effects of different levels of variability and Δ*P*_S_ in terms of gas exchange and respiratory mechanics in the short term but is intrinsically unable to investigate major clinical outcomes. The sample size is relatively low, no static measurements of respiratory mechanics were performed, and patients received heterogeneous sedation regimens that might have affected differently the respiratory drive. The population included in the study identifies a subgroup of critically ill patients meeting the criteria for mild to moderate ARDS who already received controlled or assisted mechanical ventilation for several days; however, the baseline patient characteristics were similar to those reported in a recent large observational study in patients with ARDS assisted non-invasively (Bellani et al., 2017). These patients with established respiratory failure, thus, possibly consolidated lung areas, might not benefit from the cyclic recruitment effect of vPSV, while patients with early ARDS might respond differently. However, the role of spontaneous breathing in the early management of ARDS is still unclear. This study could neither elucidate the mechanisms of the neural responses of the patients to variability nor the neuromuscular coupling of the respiratory muscles.

## Conclusion

In our cohort of patients with mild to moderate ARDS, vPSV did not improve gas exchange at different levels of variability and pressure support. Compared with PSV, vPSV increased the variability of *V*_T_, but not when low levels of variability were used in conjunction with lower pressure support. Moreover, vPSV did not exert a clinically relevant effect on the average inspiratory effort and work of breathing.

## Data Availability Statement

The raw data supporting the conclusions of this article will be made available by the authors, without undue reservation.

## Ethics Statement

The study was approved by the Local Ethical Review Board (Comitato Etico Aziendale Policlinico San Martino protocol no. 1052/12) and prospectively registered on clinicaltrials.gov (study identifier: NCT01683669). According to the local ethical requirements, the next of kin provided written informed assent, followed by delayed written consent from patients in case of recovery of consciousness.

## Author Contributions

LB takes responsibility for the integrity of data. LB, PP, MV, and MG designed the study. LB, YS, MF, AD'O, DD'A, PRa, and IB conducted the study. LB, LM, MF, RH, AD'O, and CR analyzed the data. LB, MS, PP, PRo, and MG wrote the manuscript. All authors read and approved the final version of the manuscript.

## Funding

This study was performed with institutional funding only.

## Conflict of Interest

MG was granted a patent on the variable pressure support ventilation mode of assisted ventilation (noisy PSV), which has been licensed to Dräger Medical AG (Lübeck, Germany). The remaining authors declare that the research was conducted in the absence of any commercial or financial relationships that could be construed as a potential conflict of interest.

## Publisher's Note

All claims expressed in this article are solely those of the authors and do not necessarily represent those of their affiliated organizations, or those of the publisher, the editors and the reviewers. Any product that may be evaluated in this article, or claim that may be made by its manufacturer, is not guaranteed or endorsed by the publisher.

## Abbreviations

PSV: pressure support ventilation
ARDS: acute respiratory distress syndrome
vPSV: variable pressure support ventilation
Δ*P*_S_: pressure support level
ICU: intensive care unit
PEEP: positive end-expiratory pressure
PBW: predicted body weight
SAPS: simplified acute physiology score
SOFA: sequential organ failure assessment score
RASS: richmond agitation-sedation scale
PSV_0%_: pressure support ventilation with no variability
vPSV_15%_: variable pressure support ventilation with 15% CV variability
vPSV_30%_: variable pressure support ventilation with 30% CV variability
*V*_T_: tidal volume
PTP: esophageal pressure-time product
Δ*P*_es_: esophageal pressure swings
*P*_L_: peak transpulmonary pressure
PSV_BL_: pressure support ventilation with no variability and baseline Δ*P*_S_ as per clinical indication
vPSV_BL_: variable pressure support with variability set to achieve ±5 cmH_2_O and baseline Δ*P*_S_ as per clinical indication
PSV_−5_: pressure support ventilation with no variability and Δ*P*_S_ reduced by 5 cmH_2_O from the baseline value
vPSV_−5_: variable pressure support ventilation with variability set to achieve ±5 cmH_2_O and Δ*P*_S_ reduced by 5 cmH_2_O from the baseline value.
