# Supplementary material for: Effects of Different Levels of Variability and Pressure Support Ventilation on Lung Function in Patients With Mild–Moderate Acute Respiratory Distress Syndrome
Source: Front Physiol. 2021 Oct 22;12:725738. doi: 10.3389/fphys.2021.725738 (PMC8569865; doi:10.3389/fphys.2021.725738)
Supplement: Supplementary file 1 [file Data_Sheet_1.DOCX]

**Effects of different levels of variability and pressure support ventilation on lung function in mild-moderate ARDS patients**

Online Supplement

Lorenzo Ball, Yuda Sutherasan, Martina Fiorito, Antonella Dall’Orto, Lorenzo Maiello, Maria Vargas, Chiara Robba, Iole Brunetti, Davide D’Antini, Pasquale Raimondo, Robert Huhle, Marcus J. Schultz, Patricia RM Rocco, Marcelo Gama de Abreu, Paolo Pelosi

Table of Contents

[eFIGURE 1 – Latin square used for randomisation, Block 1 2](#_Toc26345550)

[eFIGURE 2 – Latin square used for randomisation, Block 2 2](#_Toc26345551)

[eFIGURE 3 – Pressure support at different time-points 3](#_Toc26345552)

[eFIGURE 4 – Tidal volume at different time-points 4](#_Toc26345553)

[eFIGURE 5 – Mean airway pressure at different time-points 5](#_Toc26345554)

[eFIGURE 6 – End-expiratory airway pressure at different time-points 6](#_Toc26345555)

[eFIGURE 7 – Respiratory rate at different time-points 7](#_Toc26345556)

[eFIGURE 8 – Esophageal pressure-time product at different time-points 8](#_Toc26345557)

[eFIGURE 9 – Delta esophageal pressure at different time-points 9](#_Toc26345558)

[eFIGURE 10 – Peak transpulmonary pressure at different time-points 10](#_Toc26345559)

[eFIGURE 11 – Tinsp/Ttot at different time-points 11](#_Toc26345560)

# eFIGURE 1 – Latin square used for randomisation, Block 1

| **PSV_0%_** | **vPSV_15%_** | **vPSV_30%_** |
| --- | --- | --- |
| **vPSV_15%_** | **vPSV_30%_** | **PSV_0%_** |
| **vPSV_30%_** | **PSV_0%_** | **vPSV_15%_** |

**eFigure 1** – Latin square used for randomisation during the first ventilation block. PSV_0%_: conventional PSV with no variability, vPSV_15%_: variable PSV with variability set to 15% CV, vPSV_30%_: variable PSV with variability set to 30% CV; CV: coefficient of variation; PSV: pressure support ventilation.

# eFIGURE 2 – Latin square used for randomisation, Block 2

| **PSV_BL_** | **vPSV_BL_** | **PSV_-5_** | **vPSV_-5_** |
| --- | --- | --- | --- |
| **vPSV_BL_** | **vPSV_-5_** | **PSV_BL_** | **PSV_-5_** |
| **PSV_-5_** | **PSV_BL_** | **vPSV_-5_** | **vPSV_BL_** |
| **vPSV_-5_** | **PSV_-5_** | **vPSV_BL_** | **PSV_BL_** |

**eFigure 1** – Latin square used for randomisation during the second ventilation block. PSV_BL_: conventional PSV with no variability and ∆P_S_ set to the baseline value; vPSV_BL_: variable PSV with variability set individually to ± 5 cmH_2_O and ∆P_S_ set to the baseline value; PSV_-5_: conventional PSV with no variability and ∆P_S_ set to the baseline value minus 5 cmH_2_O; vPSV_-5_: variable PSV with variability set individually to ± 5 cmH_2_O and ∆P_S_ set to the baseline value minus 5 cmH_2_O.

# eFIGURE 3 – Pressure support at different time-points

# eFIGURE 4 – Tidal volume at different time-points

# eFIGURE 5 – Mean airway pressure at different time-points

# eFIGURE 6 – End-expiratory airway pressure at different time-points

# eFIGURE 7 – Respiratory rate at different time-points

# eFIGURE 8 – Esophageal pressure-time product at different time-points

# eFIGURE 9 – Delta esophageal pressure at different time-points

# eFIGURE 10 – Peak transpulmonary pressure at different time-points

# eFIGURE 11 – Tinsp/Ttot at different time-points
